# Effectiveness of Aromatherapy on Ameliorating Fatigue in Adults: A Meta-Analysis

**DOI:** 10.1155/2022/1141411

**Published:** 2022-04-13

**Authors:** Qiuting Wang, Lin Wei, Yueming Luo, Lijun Lin, Chong Deng, Ping Hu, Lijia Zhu, Yangchen Liu, Meizhen Lin

**Affiliations:** ^1^The Second Clinical College of Guangzhou University of Chinese Medicine, Guangzhou, Guangdong, China; ^2^The Second Affiliated Hospital of Guangzhou University of Chinese Medicine, Guangzhou, Guangdong, China; ^3^The Fourth Clinical Medical College of Guangzhou University of Chinese Medicine, Shenzhen Traditional Chinese Medicine Hospital, Shenzhen, China; ^4^Basic Medical Science College of Guangzhou University of Chinese Medicine, Guangzhou, Guangdong, China; ^5^The Nursing College of Hunan University of Chinese Medicine, Changsha, Hunan, China

## Abstract

**Background:**

Fatigue is a common symptom in adults that may cause physical and psychological problems and reduce quality of life. Aromatherapy could possibly provide relief for those suffering from fatigue. Here, we evaluated the effect of aromatherapy on fatigue in adults.

**Methods:**

We searched the PubMed, Embase, Cochrane Library, Web of Science, China National Knowledge Infrastructure, Chinese Biomedical Literature, SinoMed, Wanfang, and Chinese Scientific Journal Database databases for randomized controlled trials of aromatherapy treatment for fatigue in adults from their inception to June 2021. Two reviewers searched independently, extracted the characteristics of the studies, and assessed the risk of bias using the Cochrane risk-of-bias tool and Stata v. 14.0.

**Results:**

Nineteen studies were included in this systematic review. Aromatherapy had a significant effect on fatigue (standardized mean difference −0.64, 95% confidence interval−1.14, −0.15, I^2^ 94.4%, *P* < 0.001). Subgroup analysis according to aromatic type, substance, frequency, treatment duration, intervention, outcomes measurement, and population type showed that aromatherapy had a significantly greater effect in the intervention group, compared to the control group. Funnel plots and Egger's test indicated no significant publication bias.

**Conclusion:**

Our results suggest that aromatherapy ameliorates fatigue in adults who suffer from chronic diseases. A rigorous intervention program and larger randomized controlled trials are needed.

## 1. Background

Fatigue is a subjective feeling of tiredness, weakness, or lack of energy and motivation [1]. It is distressing and highly prevalent in adults, particularly in those diagnosed with cancer, receiving hemodialysis or suffering from chronic diseases. It has no specific mechanism and can occur at any disease stage [1]. Fatigue is related to an elevated incidence of physical and mental diseases (such as cardiovascular disease, anxiety, depression, and sleep disorders), which reduce quality of life and prolong hospitalization [2, 3]. Approximately 5–40% of patients experience fatigue from hospitalization and follow-up visits, and >50% of patients with chronic hepatitis C virus infection and 60–97% of those undergoing hemodialysis feel fatigue [4, 5].

Aromatherapy is the application of plant essential oils or herbal essences by inhalation, massage, or compression to alleviate a symptom or disease [6]. As a nonpharmacological, complementary, and alternative modality, aromatherapy is economical and has fewer adverse effects compared with Western medicine. The United States federal government funds aromatherapy research with $30.2 billion annually [7]. Aromatherapy can improve symptoms such as sleep problems, pain, chronic fatigue, anxiety, depression, stress, and postoperative nausea and vomiting [8, 9].

Most RCTs show the significant effect of aromatherapy on fatigue, but others have reported discrepant findings. Moreover, reviews have verified the efficacy of complementary and alternative therapies, such as acupuncture, moxibustion, Tai Chi, and acupressure on fatigue [10]. Hence, this meta-analysis evaluated the evidence and estimated effects of aromatherapy on fatigue in adults.

## 2. Methods

This systematic review and meta-analysis was conducted according to the Preferred Reporting Items for Systematic Reviews and Meta-Analyses (PRISMA) guidelines [11] and is registered at the International Prospective Register of Systematic Reviews (no. CRD42021268038).

### 2.1. Search Strategy

Two reviewers (Q.T. Wang and P. Hu) independently and systematically searched for relevant studies in the PubMed, Embase, Cochrane Library, Web of Science, China National Knowledge Infrastructure, Chinese Scientific Journal Database, Chinese Biomedical Literature, and Wanfang databases. A manual search was not performed in this study. The search terms comprised aromatherapy (or aroma therapy, aromatherapies, etc.) and fatigue (or lassitude). The search process had no date or language restrictions. Taking PubMed as an example, we used the following search parameters: (aromatherapy [Mesh]) OR (((((Aromatherapies [Title/Abstract])) OR (Aroma Therapy [Title/Abstract])) OR (Aroma Therapies [Title/Abstract])) OR (Therapy, Aroma [Title/Abstract])) OR (Therapies, Aroma [Title/Abstract]) AND ((fatigue [Mesh Terms])) OR (Lassitude[Title/Abstract]). Both mesh and non-mesh terms were included.

### 2.2. Eligibility Criteria

Inclusion and exclusion criteria were determined according to the participants, interventions, comparisons, outcomes, and study design principles. The inclusion criteria were (1) adults (≥18 years) diagnosed with fatigue regardless of race, sex, disease type, or disease duration; (2) the treatment group received aromatherapy, the details of which were described; (3) the control group received a placebo, regular care, or no treatment; (4) the degree of fatigue was regarded as the primary or secondary outcome and was estimated by a fatigue scale, such as the Fatigue Severity Scale, Multidimensional Fatigue Inventory, Brief Fatigue Inventory, or a Visual Analog Scale; and (5) the included studies were randomized controlled trials (RCTs) published in any language.

### 2.3. Exclusion Criteria

We excluded trials of chronic fatigue syndrome, defined as persistent fatigue over 6 months with multisystem disorders [9]. RCTs, quasi-randomized trials, and parallel trials were included. Trials that did not report outcomes or included incomplete data were excluded.

### 2.4. Study Selection

We searched the articles that met the inclusion criteria and created a database using EndNote v. 9.0 software. According to the PRISMA flow diagram [11], we removed duplicate studies, screened the titles and abstracts, and finally browsed the full text to identify relevant RCTs.

### 2.5. Data Extraction

Two reviewers (Q.T. Wang and L.J. Zhu) extracted the data, and another reviewer (Y.C. Liu) checked for accuracy. Discrepancies were resolved by discussion until a consensus was achieved. Information collected from the trials consisted of the first author, publication year, country, participants' details (e.g., age, sample size, type of disease), interventions (*e*.g., aromatic mode, dosage, duration and frequency), and outcome metrics [12]. If necessary, one reviewer (Q.T. Wang) contacted the author to obtain missing information.

### 2.6. Risk of Bias Assessment

Using the Cochrane Collaboration Risk of Bias Tool, the risk of bias was assessed as low risk, high risk, or unclear by two reviewers (Q.T. Wang and Y.C. Liu) separately [13]. We ranked risk based on the following seven domains: random sequence generation, allocation concealment, blinding of participants and personnel, blinding of outcome assessment, incomplete outcome data, selective reporting, and other biases. Disagreements were resolved by discussion until a consensus was achieved.

### 2.7. Statistical Analysis

We analyzed the data using Stata v. 14.0 software. Continuous data are presented as means and standard deviations. Outcomes were synthesized as standardized mean differences and 95% confidence intervals using a random-effects model. Subgroup analyses were performed to identify sources of heterogeneity, including aromatic type, substance, frequency and treatment duration, the control intervention, outcomes assessment, and type of population. A sensitivity analysis was conducted to evaluate the stability of the results and whether the meta-analysis results were affected by any of the individual trials. If heterogeneity was significant (I ^2^ ≥ 50% or *p* < 0.10) [14], we used the random-effects model or screened the included studies one by one to identify the influential factors. A fixed-effects model was applied in the meta-analysis if there was no significant heterogeneity. *p* < 0.05 was considered indicative of statistical significance. Funnel plots and Egger's test were used to assess publication bias.

## 3. Results

### 3.1. Literature Search

**Figure 1:**
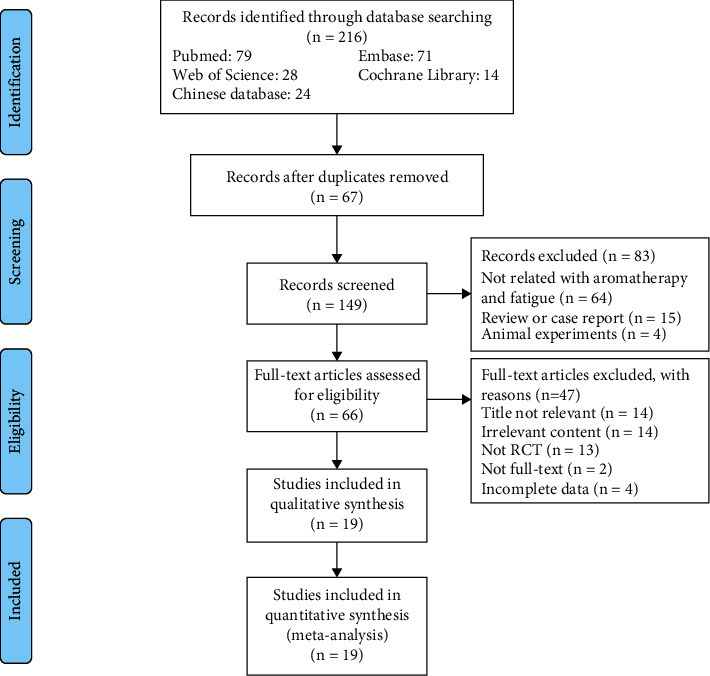
Flow diagram of trial selection.

We identified 216 studies in the electronic databases, among which 19 were included in the analysis after removing 67 duplicates. One hundred forty-nine articles were excluded after screening the title and abstract. Among the remaining 66 articles, 47 were excluded due to an irrelevant title (*n* = 14), irrelevant abstract (*n* = 14), non-RCT (*n* = 13), no full-text (*n* = (2), or incomplete data (*n* = 4). Finally, 19 studies were eligible for the meta-analysis. The selection process is shown in Figure 1.

### 3.2. Study Characteristics

A total of 1381 participants were included in this meta-analysis. The 19 studies were published in Iran [15–22], Turkey [23–26], Korea [27], South Korea [28, 29], Japan [30, 31], China [32], or the United States [33] from 2012 to 2021. The essential oils used as interventions were lavender, citrus, and mixed essential oils. The control groups received routine care or a placebo (distilled water or vegetable juice). The oil dose was 2/3/5/8/20 drops, and 12 and 7 comparisons used inhalation and massage, respectively. The intervention frequency was two, three, or four times per week, or one to two times per day, or every other day. The treatment duration ranged from 1 or 2 days to 8 weeks. The primary outcome was fatigue as measured by the Fatigue Severity Scale (FSS), Brief Fatigue Inventory (BFI), Multidimensional Fatigue Inventory (MFI), Numeric Rating Scale (NRS), Visual Analog Scale (VAS), and so on. The characteristics of the included trials are listed in Table 1.

### 3.3. Risk of Bias

**Figure 2:**
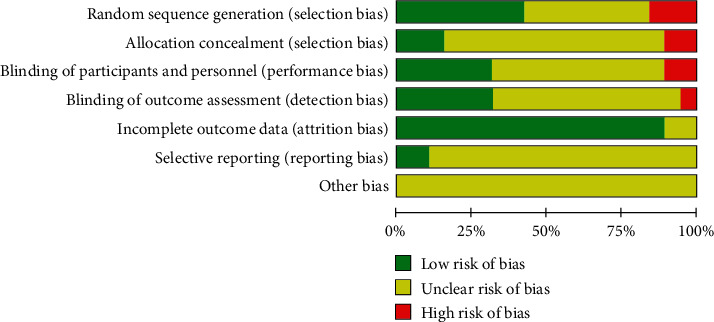
Risk of bias graph.

**Figure 3:**
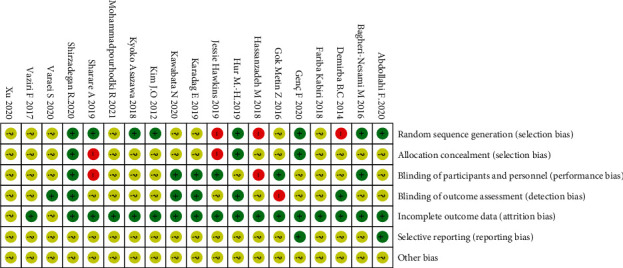
Risk of bias summary.

Eight trials were considered low risk because they applied random sequence generation. Three trials did not mention randomization and thus were ranked as high risk. Only three studies described the process of allocation concealment. Two trials were high risk due to the use of an improper method of allocation concealment. Six studies were assessed as low risk because they had single or triple blinding; by contrast, one had incomplete blinding and thus was ranked as high risk. One trial was high risk because the blinding was broken, and six trials were low risk. Two trials were assessed as unclear risk and the others as low risk. Two trials were low risk because they explained the outcome report; the others were unclear risk. No other type of bias was detected (Figures 2 and 3).

### 3.4. Overall Effect of Aromatherapy

**Figure 4:**
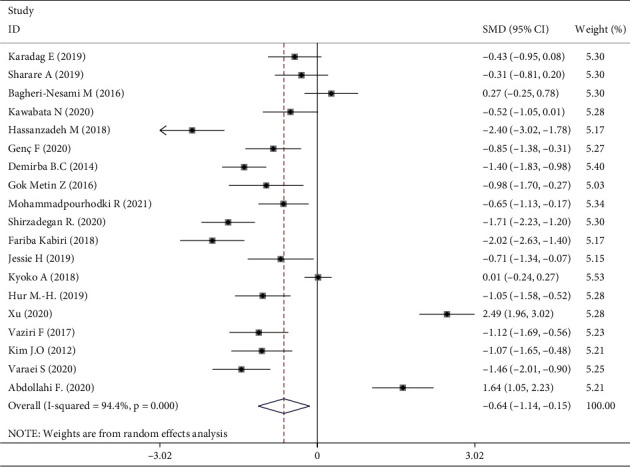
Meta-analysis of aromatherapy group versus control group on fatigue in adults.

The 1381 participants were divided into intervention (*n* = 691) and control (*n* = 690) groups. Using a random-effects model, the intervention group showed a significant effect on fatigue compared with the control group (standardized mean difference, −0.64; 95% confidence interval −1.14, −0.15). Aromatherapy significantly relieved fatigue, but the studies showed high heterogeneity (I^2^ 94.4%, *P* < 0.001) (Figure 4).

### 3.5. Subgroup Analysis

**Figure 5:**
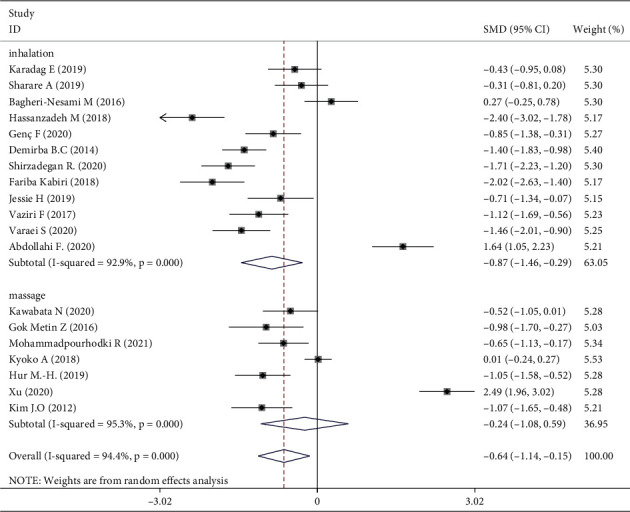
Subgroup analysis of aromatic delivery mode.

**Figure 6:**
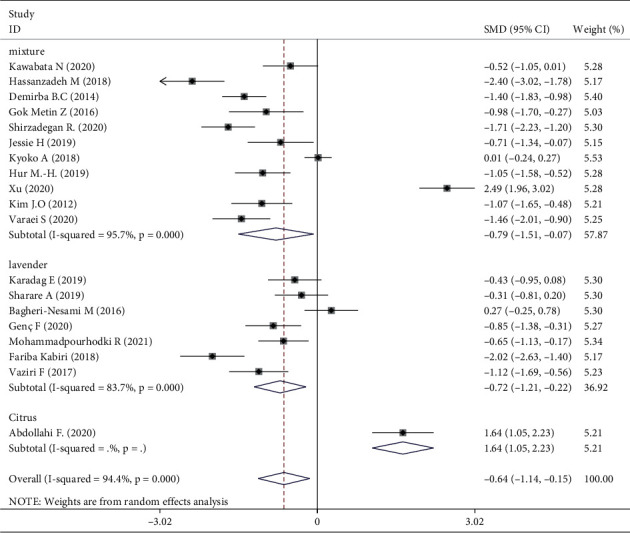
Subgroup analysis of substance.

**Figure 7:**
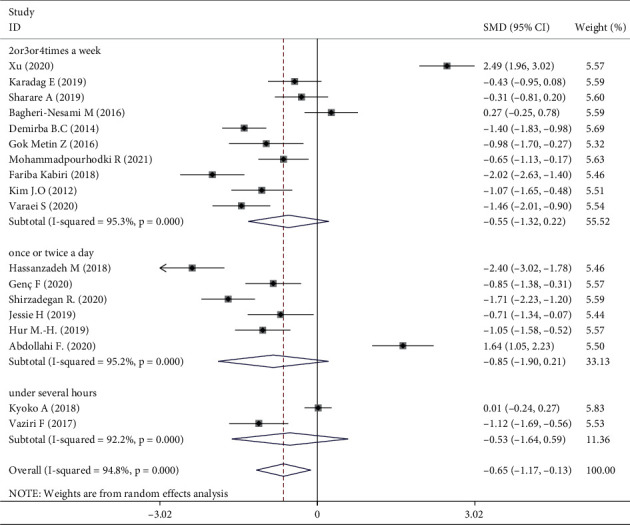
Subgroup analysis of frequency.

**Figure 8:**
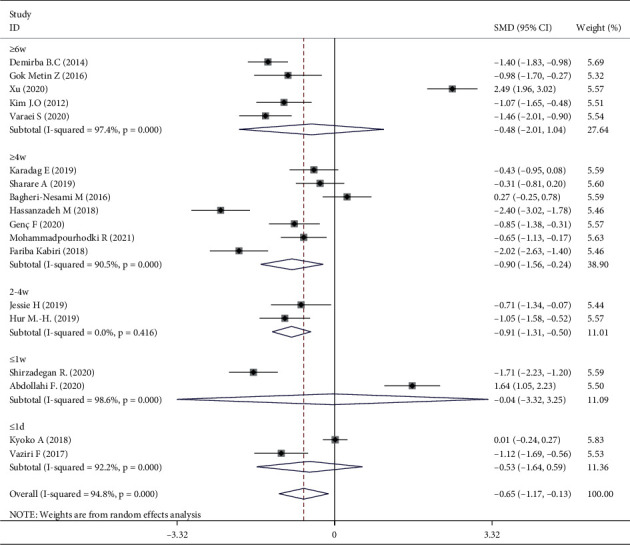
Subgroup analysis of treatment duration.

**Figure 9:**
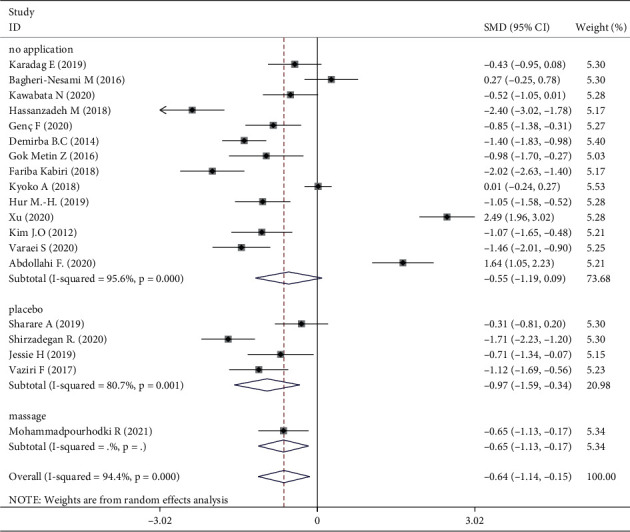
Subgroup analysis of control intervention.

**Figure 10:**
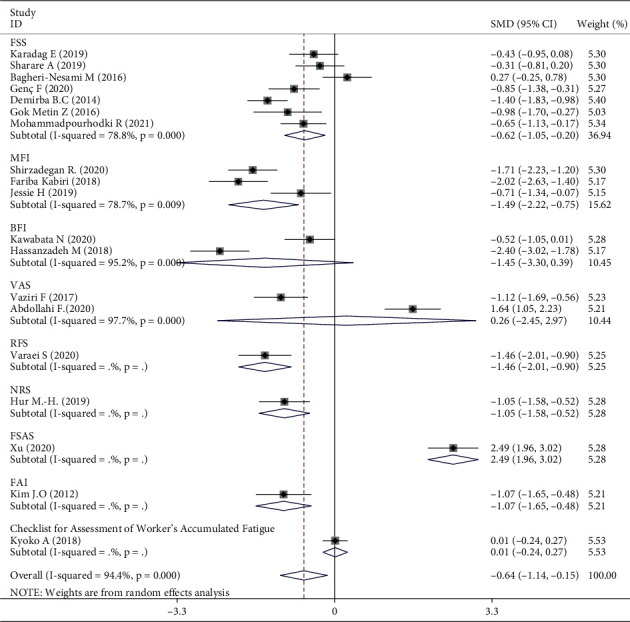
Subgroup analysis of outcomes measurement.

**Figure 11:**
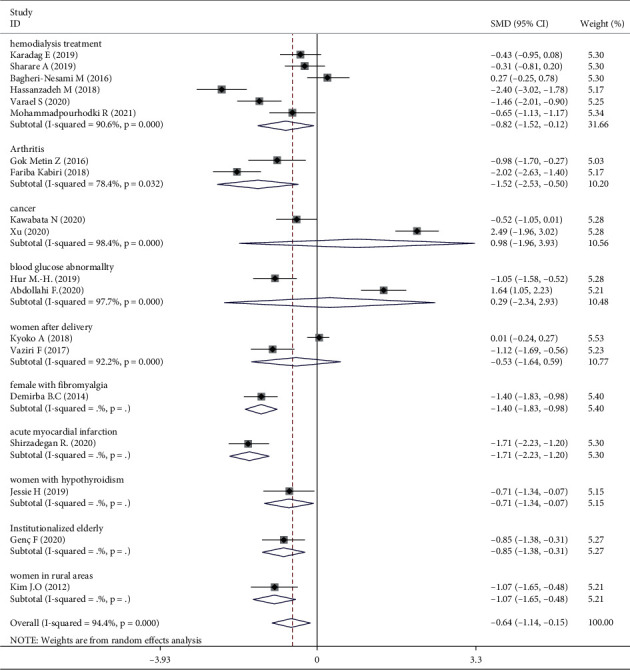
Subgroup analysis of type of population.

Meta-analysis of three trials [16, 30, 31] showed that aromatherapy did not ameliorate fatigue in adults. Therefore, we conducted subgroup and sensitivity analyses to estimate the effect of aromatherapy on fatigue. Considering the high heterogeneity among the studies, we performed subgroup analyses according to aromatic delivery mode, substance, frequency, treatment duration, control intervention, outcome measurement, and type of population. The results suggested that aromatherapy significantly relieved fatigue compared with the control group, irrespective of the aromatic delivery mode (Figure 5). Regarding aromatic substances, lavender essential oil and mixed oils were effective for fatigue, whereas citrus oil was not (Figure 6). Regarding aromatic frequency, there were differences in efficacy between the intervention and control groups. However, when aromatherapy was administered two, three, or four times weekly, once or twice daily, for several hours, the heterogeneity was significant (Figure 7). The heterogeneity of studies with treatment durations of ≥6 weeks and ≤1 week was higher than that of the other studies (Figure 8). The effect of aromatherapy did not differ between the intervention and control groups when the treatment period lasted less than 1 day. Additionally, aromatherapy for 2–4 weeks or 4 weeks showed considerable effectiveness in the intervention group, regardless of the control intervention used (placebo control, massage, or no application) (Figure 9). For the outcome assessment and type of population, the subgroup analysis showed a significant difference between the intervention and control groups on fatigue (Figures 10, 11).

### 3.6. Publication Bias and Sensitivity Analysis

**Figure 12:**
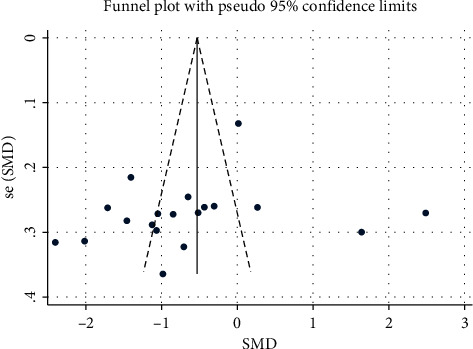
Funnel plot.

**Figure 13:**
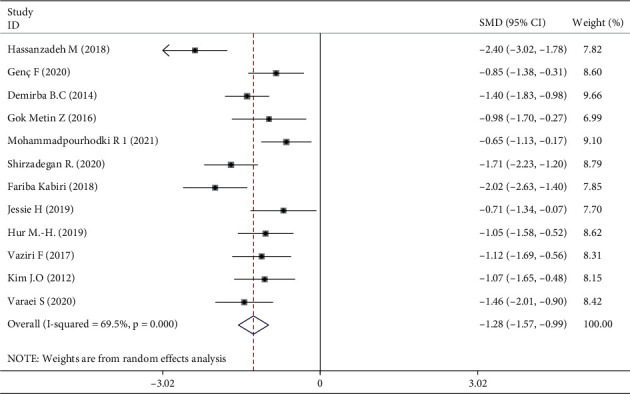
Sensitivity analysis.

The funnel plot was symmetrical, so deviation was associated with trial methodology (Figure 12). Egger's test (*P*=0.621) showed no significant publication bias in terms of the effectiveness of aromatherapy on fatigue. A sensitivity analysis in which trials were excluded one by one [16, 18, 21, 23, 30–32] did not significantly alter the meta-analysis results (I^2^ 69.5%, *P* < 0.001) (Figure 13).

## 4. Discussion

Aromatherapy is reportedly effective for sleep disorders, anxiety, stress, labor pain, and postoperative nausea and vomiting [34–38]. It is an inexpensive, nonpharmacological treatment, with few side effects and is convenient to administer in the home or clinic [38, 39]. Adverse effects are rare, although infrequent occurrences of allergy and drowsiness were reported by a small RCT (*N* = 7) [40]. Essential oils are derived from the petals, flowers, stems, leaves, needles, rinds, fruits, roots, and rhizomes of lavender, rose, orange, lemon, citrus, almond, peppermint, ginger, and so on. The oil comprises multiple natural volatile organic compounds because it is produced through distillation, extraction, or concentration from the plants by steam or a mechanical cold press [7, 41]. Its therapeutic effects are based on the systemic functions of the body (working like a drug or enzyme), which is used to trigger reflexive effects to generate a positive emotion [41].Although the mechanism by which aromatherapy relieves fatigue is unclear, it is said that aromatherapy activates the limbic system, interacting with the cerebral cortex to regulate the individual's emotion and visceral functions, such as the heart rate, respiration, blood pressure, blood flow, muscle tension, body temperature, pupil dilation, and hormonal levels [7, 23]. Aromatherapy can be delivered by inhalation, massage, compression, or foot baths [42]. Research indicates that aromatic inhalation stimulates the olfactory nerve cells and the integumentary and lymphatic systems, triggering the release of neurotransmitters, such as endorphins, peptides, enkephalin, serotonin, and noradrenaline, which can enhance wellbeing and relaxation, thus reducing chronic stress [7, 20, 34, 39]. Smell is mainly governed by the brain's limbic system, which is also associated with emotions, feeling, and behavior. Massage is thought to play a role in the skin, blood, and lymphatic systems. Moreover, aromatic massage promotes the absorption of essential oils and activates mental and physiological responses, including an immune system response [7, 19]. Additionally, aromatic massage not only facilitates the absorption of essential oil through the skin but also keeps the skin warm and relaxes the body [19].

A meta-analysis of the effects of aromatherapy on insomnia concluded that inhalation is more effective than massage. However, a systematic review showed that aromatic massage is more beneficial than aromatic inhalation for depressive symptoms [43]. One RCT showed that the effect of aromatherapy massage is stronger than that of inhalation on fatigue in hemodialysis patients [17]. Our subgroup analysis of aromatic delivery showed no significant difference between inhalation and massage. A single essential oil is more beneficial for sleep than mixed oils [34]. Conversely, a meta-analysis concluded that multiple aromatic oils were more effective for fatigue in patients receiving hemodialysis [43]. Indeed, our subgroup analysis showed that both lavender and mixed oils relieve fatigue. Citrus, sweet almond, and orange oils showed no significant effect, possibly due to the low quality of the studies [18, 21]. In an RCT of lavender and orange essential oils for fatigue in hemodialysis patients [18], the outcome showed no significant difference between the lavender and orange groups. Another study observed no significant difference between lavender and citrus aurantium essential oils used in massage to relieve fatigue [19].

Aromatherapy duration for 2–4 weeks or >4 weeks was effective, as demonstrated in one of the included studies [18]. However, the RCTs used different treatment durations. One study suggested that aromatherapy administrated for <2, 2–4, or >4 weeks had similar levels of efficacy [14]. There were differences in efficacy between the intervention and control groups for aromatherapy administered two, three, or four times weekly, whereas application once or twice daily showed no significant difference in efficacy between the intervention and control groups. The frequency of aromatherapy influenced efficacy with regard to fatigue from the subgroup analysis. The results showed no obvious effect on fatigue for whole treatments lasting several hours [28, 31].

For the subgroup analysis of outcome assessment and type of population, the effect of aromatherapy on fatigue was significant in a comparison between the intervention groups and the controls. There are various scales to measure people's fatigue, according to different diseases. Typically, the outcome assessment tools are chosen to fit the specific purpose of the fatigue measurement in the group participants. The scales in this study had been assessed in accordance with their content, constructive validity, and reliability and were used to measure fatigue in response to aromatherapy, as well as detect changes in disease progression over time. However, it is unclear as to the specific assessment scale/s used for fatigue level, thus making comparison between studies difficult [44]. Similarly, different types of population experience varying degrees of fatigue, and each has its own scale. The participants were female in six RCTs [26, 28, 29, 31–33] included in our analysis, and many of the clinical trials focused on women's health in using aromatherapy, for example, for menopausal symptoms, delivery, and dysmenorrhea [45]. However, there was no evidence that supported the prevalence or effects of aromatherapy use with respect to gender. We can only speculate as to why many of the studies included female participants; there was no clear reason for this in relation to the proportion suffering from chronic disease or having a stronger response to aromatherapy.

Three RCTs indicated that aromatherapy had no effect on fatigue. Subgroup analyses suggested significant heterogeneity in eight groups (Figures 5–11). A sensitivity analysis showed that seven trials were likely sources of high heterogeneity, due to being of low quality or having design deficiencies. Overall, there was no clear explanation for the high heterogeneity; thus, more hard evidence of the effects of aromatherapy on fatigue is needed.

### 4.1. Strengths and Limitations

We used rigorous inclusion criteria to generate reliable and objective outcomes, resulting in analysis of 19 trials. Furthermore, most previous studies focused on inhalation aromatherapy [34], but we considered both inhalation and massage aromatherapy. Finally, we used several scales to evaluate fatigue, resulting in robust results.

This study had several limitations. First, the included trials had methodological deficiencies. Only three trials performed both random sequence generation and allocation concealment. Six trials implemented blinding of participants and personnel (single or triple), six applied blinding of outcome assessments, and two trials had unclear bias and incomplete outcome data. Second, the high heterogeneity may be related to the diversity of medical conditions (which may affect fatigue scores) evaluated in the included RCTs. However, most of the trials used similar methodologies. Third, we did not evaluate the optimal treatment duration, which could influence effectiveness.

## 5. Conclusion

Our results showed that aromatherapy is effective for relieving fatigue in adults who suffer from chronic diseases, such as cancer, arthritis, hypothyroidism, diabetes, renal disease, and so on. However, several trials showed that aromatherapy did not ameliorate fatigue; this could be the result of treatment duration or study quality. Additionally, a scientific aromatherapy program and further high-quality RCTs are necessary to assess the effect of aromatherapy on fatigue.

## Figures and Tables

**Table 1:** Characteristics of the included RCTs.

The characteristics of the included studies in the meta-analysis											
Author (Year)	Country	Population characteristics	Age (y)	Sample Size (intervention/control)	Interventions		Aromatic type	Aromatic frequency	Dosage	Treatment duration	Outcome measurement
					Intervention group	Control group					
Karadag E 2019	Turkey	Received hemodialysis treatment	18–65	30/30 60	2% lavender oil	Routine care	Inhalation	2 or 3 times a week	2 drops	30 days	Fatigue Severity Scale (FSS)
Sharare A 2019	Iran	Received hemodialysis treatment	18–65	30/30 60	Lavender essential oil	Placebo	Inhalation	3 or 4 times a week	5 drops	Unknown	Fatigue Severity Scale (FSS)
Bagheri-Nesami M 2016	Iran	Received hemodialysis treatment	≥18	29/30 59	5% lavender essential oil	Routine care	Inhalation	3 times a week	3 drops	4 weeks	Fatigue Severity Scale (FSS)
Kawabata N 2020	Japan	Diagnosed with advanced cancer	≥18	27/30 57	Mixture oils	Routine care	Massage	Unknown	Unknown	Unknown	Brief Fatigue Inventory (BFI)
Hassanzadeh M 2018	Iran	Receiving hemodialysis treatment	20–65	35/35 70	a mixture of 5% lavender essential oil and sweet almond oil	Routine care	Inhalation	Twice a day	2 drops	4 weeks	Brief Fatigue Inventory (BFI)
Genç F 2020	Turkey	The institutionalized elderly	≥65	30/29 59	3% lavender oil	Routine care	Inhalation	Once a day	2 drops	a month	Fatigue Severity Scale (FSS)
Demirba BC 2014	Turkey	Female patients with fibromyalgia	Unknown	54/54 108	Mixture oils	Routine care	Inhalation	Every other day	Unknown	6 weeks	Fatigue Severity Scale (FSS)
Gok Metin Z 2016	Turkey	Diagnosis of Rheumatoid arthritis	≥18	17/17 34	5% mixture oils	Routine care	Massage	3 times a week	20 drops	6 weeks	Fatigue Severity Scale (FSS)
Mohammadpourhodki R 2021	Iran	Receiving hemodialysis treatment	18–65	35/35 70	1.5% lavender essential oil	Massage	Massage	3 times a week	10 to 15 cubic centimeter	Unknown	Fatigue Severity Scale (FSS)
Shirzadegan R 2020	Iran	Diagnosis of acute myocardial infarction	18–60	40/40 80	Mixture oils	Placebo	Inhalation	Twice a day	3 drops	2 days	Multidimensional Fatigue Inventory (MFI)
Fariba Kabiri 2018	Iran	Diagnosis of knee osteoarthritis	40–60	31/31 62	Lavender oil	Routine care	Inhalation	Every other day	2 drops	one month	Multidimensional Fatigue Inventory (MFI)
Jessie Hawkins 2019	USA	Women diagnosed with hypothyroidism	18–55	21/20 41	Mixture oils	Placebo	Inhalation	Once a day	3 drops	2 weeks	Multidimensional Fatigue Symptom Inventory (MFSI)
Kyoko Asazawa 2018	Japan	Women in early postpartum period	Average 30	115/114 229	2% mixture oils	No application	Massage	Unknown	Unknown	1 session	Self-Diagnosis Checklist for Assessment of Worker's Accumulated Fatigue
Hur M-H 2019	South Korea	Prediabetic women	40–65	31/31 62	3% mixture oils	Routine care	Massage	Once a day	20 drops	2 weeks	Numeric Rating Scale (NRS)
Xu 2020	China	Diagnosis of certain cancer	Unknown	49/49 98	Mixture oils	Routine care	Massage	2 times a week	1–4 drops	8 weeks	Fatigue Self-Assessment Scale (FSAS)
Vaziri F 2017	South Korea.	Women after normal vaginal delivery	18–35	29/27 56	Lavender oil	Placebo	Inhalation	6hours	5 drops	The first 24h after delivery	Visual Analog Scale (VAS)
Kim JO 2012	*Korea.*	Women in rural areas	Unknown	26/26 52	Mixture oils	Routine care	Massage	3 times a week	Unknown	6 weeks	Fatigue Assessment Instrument (FAI)
Varaei S 2020	Iran	Receiving hemodialysis treatment	Unknown	32/32 64	Mixture oils of lavender and sweet orange	Routine care	Inhalation	3 times a week	2 drops	8 weeks	Rhoten Fatigue Scale (RFS)
Abdollahi F 2020	Iran	Type 2 diabetic patients	30–65	30/30 60	Citrus(bitter orange)	Routine care	Inhalation	Once a day	8 drops	3 days	Visual Analog Scale (VAS)

## Abbreviations

RCTs: Randomized controlled trials
RCT: Randomized controlled trial
CNKI: China National Knowledge Infrastructure
VIP: Chinese Science and Technology Periodical Database
CBM: Chinese Biomedical Literature Database
CFS: Chronic Fatigue Syndrome
CCRBT: The Cochrane Collaboration Risk of Bias Tool
CI: Confidence interval
M: Mean
SD: Standard deviation
SMD: Standard mean difference.

## Supplementary Materials

Supplementary Materials Data Table 1: meta-analysis of aromatherapy group versus control group on fatigue in adults. Data Table 2: subgroup analysis of aromatic delivery mode. Data Table 3: subgroup analysis of substance. Data Table 4: subgroup analysis of frequency. Data Table 5: subgroup analysis of treatment duration. Data Table 6: subgroup analysis of control intervention. Data Table 7: subgroup analysis of outcomes measurement. Data Table 8: subgroup analysis of type of population. Data Table 9: sensitivity analysis.
