# Supplementary material for: Effectiveness of Aromatherapy on Ameliorating Fatigue in Adults: A Meta-Analysis
Source: Evid Based Complement Alternat Med. 2022 Apr 13;2022:1141411. doi: 10.1155/2022/1141411 (PMC9020949; doi:10.1155/2022/1141411)
Supplement: Supplementary Materials — Data Table 1: meta-analysis of aromatherapy group versus control group on fatigue in adults. Data Table 2: subgroup analysis of aromatic delivery mode. Data Table 3: subgroup analysis of substance. Data Table 4: subgroup analysis of frequency. Data Table 5: subgroup analysis of treatment duration. Data Table 6: subgroup analysis of control intervention. Data Table 7: subgroup analysis of outcomes measurement. Data Table 8: subgroup analysis of type of population. Data Table 9: sensitivity analysis. [file 1141411.f1.docx]

**The Effectiveness of Aromatherapy on Ameliorating** **Fatigue in Adults: A Meta-Analysis**

Qiuting Wang^1^, Lin Wei^2^, Yueming Luo^3^, Lijun Lin^2^, Chon Deng^4^, Ping Hu^1^, Lijia Zhu^5^, Yangchen Liu^2*^,Meizhen Lin^2*^

^1^The Second Clinical College of Guangzhou University of Chinese Medicine, Guangzhou, Guangdong, China.

^2^The Second Affiliated Hospital of Guangzhou University of Chinese Medicine, Guangzhou, Guangdong, China.

^3^The Fourth Clinical Medical College of Guangzhou University of Chinese Medicine, Shenzhen Traditional Chinese Medicine Hospital, Shenzhen, China

^4^Basic Medical Science College of Guangzhou University of Chinese Medicine, Guangzhou, Guangdong, China.

^5^The Nursing College of Hunan University of Chinese Medicine, Changsha, Hunan, China.

| **Data table 1 Aromatherapy group versus control group on fatigue** | | | | | | | |
| --- | --- | --- | --- | --- | --- | --- | --- |
| author | year | Tsample | Tmean | Tsd | Csample | Cmean | Csd |
| Karadag E | 2019 | 30 | 35.23 | 5.21 | 30 | 38.46 | 9.12 |
| Sharare A | 2019 | 30 | 30.27 | 13.88 | 30 | 34.7 | 15.09 |
| Bagheri-Nesami M | 2016 | 29 | 42.61 | 3.45 | 30 | 41.7 | 3.39 |
| Kawabata N | 2020 | 27 | 19.6 | 19.7 | 30 | 31.8 | 26.6 |
| Hassanzadeh M | 2018 | 35 | 3.64 | 0.79 | 35 | 6.21 | 1.29 |
| Genç F | 2020 | 30 | 3.77 | 0.68 | 29 | 4.66 | 1.33 |
| Demirba B.C | 2014 | 54 | 4.55 | 0.81 | 54 | 5.66 | 0.77 |
| Gok Metin Z | 2016 | 17 | 2.94 | 1.13 | 17 | 4.41 | 1.79 |
| Mohammadpourhodki R | 2021 | 35 | 35.4 | 15.7 | 35 | 45.1 | 14.1 |
| Shirzadegan R. | 2020 | 40 | 24.4 | 15.75 | 40 | 71.14 | 35.25 |
| Fariba Kabiri | 2018 | 31 | 59.12 | 1.9 | 31 | 62.95 | 1.9 |
| Jessie H | 2019 | 21 | 0.9 | 0.76 | 20 | 1.79 | 1.63 |
| Kyoko A | 2018 | 115 | 8.7 | 6.6 | 114 | 8.6 | 7.5 |
| [Hur M.-H.](mailto:wowmhhur@nate.com) | 2019 | 31 | 5.52 | 1.36 | 31 | 7.1 | 1.64 |
| Xu | 2020 | 49 | 39.85 | 2.64 | 49 | 33.84 | 2.17 |
| Vaziri F | 2017 | 29 | 21.68 | 14.3 | 27 | 38.22 | 15.14 |
| Kim J.O | 2012 | 26 | 91.23 | 16.93 | 26 | 110.77 | 19.62 |
| Varaei S | 2020 | 32 | 4 | 1.67 | 32 | 6.22 | 1.36 |
| Abdollahi F. | 2020 | 30 | 63 | 3.93 | 30 | 56.26 | 4.28 |

| **Data table 2 Subgroup analysis of aromatic delivery mode** | | | | | | | | |
| --- | --- | --- | --- | --- | --- | --- | --- | --- |
| author | year | Tsample | Tmean | Tsd | Csample | Cmean | Csd | type |
| Karadag E | 2019 | 30 | 35.23 | 5.21 | 30 | 38.46 | 9.12 | inhalation |
| Sharare A | 2019 | 30 | 30.27 | 13.88 | 30 | 34.7 | 15.09 | inhalation |
| Bagheri-Nesami M | 2016 | 29 | 42.61 | 3.45 | 30 | 41.7 | 3.39 | inhalation |
| Hassanzadeh M | 2018 | 35 | 3.64 | 0.79 | 35 | 6.21 | 1.29 | inhalation |
| Genç F | 2020 | 30 | 3.77 | 0.68 | 29 | 4.66 | 1.33 | inhalation |
| Demirba B.C | 2014 | 54 | 4.55 | 0.81 | 54 | 5.66 | 0.77 | inhalation |
| Shirzadegan R. | 2020 | 40 | 24.4 | 15.75 | 40 | 71.14 | 35.25 | inhalation |
| Fariba Kabiri | 2018 | 31 | 59.12 | 1.9 | 31 | 62.95 | 1.9 | inhalation |
| Jessie H | 2019 | 21 | 0.9 | 0.76 | 20 | 1.79 | 1.63 | inhalation |
| Vaziri F | 2017 | 29 | 21.68 | 14.3 | 27 | 38.22 | 15.14 | inhalation |
| Varaei S | 2020 | 32 | 4 | 1.67 | 32 | 6.22 | 1.36 | inhalation |
| Abdollahi F. | 2020 | 30 | 63 | 3.93 | 30 | 56.26 | 4.28 | inhalation |
| Kawabata N | 2020 | 27 | 19.6 | 19.7 | 30 | 31.8 | 26.6 | massage |
| Gok Metin Z | 2016 | 17 | 2.94 | 1.13 | 17 | 4.41 | 1.79 | massage |
| Mohammadpourhodki R | 2021 | 35 | 35.4 | 15.7 | 35 | 45.1 | 14.1 | massage |
| Kyoko A | 2018 | 115 | 8.7 | 6.6 | 114 | 8.6 | 7.5 | massage |
| [Hur M.-H.](mailto:wowmhhur@nate.com) | 2019 | 31 | 5.52 | 1.36 | 31 | 7.1 | 1.64 | massage |
| Xu | 2020 | 49 | 39.85 | 2.64 | 49 | 33.84 | 2.17 | massage |
| Kim J.O | 2012 | 26 | 91.23 | 16.93 | 26 | 110.77 | 19.62 | massage |

| **Data table 3 Subgroup analysis of substance** | | | | | | | | |
| --- | --- | --- | --- | --- | --- | --- | --- | --- |
| author | year | Tsample | Tmean | Tsd | Csample | Cmean | Csd | oil |
| Kawabata N | 2020 | 27 | 19.6 | 19.7 | 30 | 31.8 | 26.6 | mixture |
| Hassanzadeh M | 2018 | 35 | 3.64 | 0.79 | 35 | 6.21 | 1.29 | mixture |
| Demirba B.C | 2014 | 54 | 4.55 | 0.81 | 54 | 5.66 | 0.77 | mixture |
| Gok Metin Z | 2016 | 17 | 2.94 | 1.13 | 17 | 4.41 | 1.79 | mixture |
| Shirzadegan R. | 2020 | 40 | 24.4 | 15.75 | 40 | 71.14 | 35.25 | mixture |
| Jessie H | 2019 | 21 | 0.9 | 0.76 | 20 | 1.79 | 1.63 | mixture |
| Kyoko A | 2018 | 115 | 8.7 | 6.6 | 114 | 8.6 | 7.5 | mixture |
| [Hur M.-H.](mailto:wowmhhur@nate.com) | 2019 | 31 | 5.52 | 1.36 | 31 | 7.1 | 1.64 | mixture |
| Xu | 2020 | 49 | 39.85 | 2.64 | 49 | 33.84 | 2.17 | mixture |
| Kim J.O | 2012 | 26 | 91.23 | 16.93 | 26 | 110.77 | 19.62 | mixture |
| Varaei S | 2020 | 32 | 4 | 1.67 | 32 | 6.22 | 1.36 | mixture |
| Karadag E | 2019 | 30 | 35.23 | 5.21 | 30 | 38.46 | 9.12 | lavender |
| Sharare A | 2019 | 30 | 30.27 | 13.88 | 30 | 34.7 | 15.09 | lavender |
| Bagheri-Nesami M | 2016 | 29 | 42.61 | 3.45 | 30 | 41.7 | 3.39 | lavender |
| Genç F | 2020 | 30 | 3.77 | 0.68 | 29 | 4.66 | 1.33 | lavender |
| Mohammadpourhodki R | 2021 | 35 | 35.4 | 15.7 | 35 | 45.1 | 14.1 | lavender |
| Fariba Kabiri | 2018 | 31 | 59.12 | 1.9 | 31 | 62.95 | 1.9 | lavender |
| Vaziri F | 2017 | 29 | 21.68 | 14.3 | 27 | 38.22 | 15.14 | lavender |
| Abdollahi F. | 2020 | 30 | 63 | 3.93 | 30 | 56.26 | 4.28 | Citrus |

| **Data table 4 Subgroup analysis of frequency** | | | | | | | | |
| --- | --- | --- | --- | --- | --- | --- | --- | --- |
| author | year | Tsample | Tmean | Tsd | Csample | Cmean | Csd | frequency |
| Xu | 2020 | 49 | 39.85 | 2.64 | 49 | 33.84 | 2.17 | 2or3or4times a week |
| Karadag E | 2019 | 30 | 35.23 | 5.21 | 30 | 38.46 | 9.12 | 2or3or4times a week |
| Sharare A | 2019 | 30 | 30.27 | 13.88 | 30 | 34.7 | 15.09 | 2or3or4times a week |
| Bagheri-Nesami M | 2016 | 29 | 42.61 | 3.45 | 30 | 41.7 | 3.39 | 2or3or4times a week |
| Demirba B.C | 2014 | 54 | 4.55 | 0.81 | 54 | 5.66 | 0.77 | 2or3or4times a week |
| Gok Metin Z | 2016 | 17 | 2.94 | 1.13 | 17 | 4.41 | 1.79 | 2or3or4times a week |
| Mohammadpourhodki R | 2021 | 35 | 35.4 | 15.7 | 35 | 45.1 | 14.1 | 2or3or4times a week |
| Fariba Kabiri | 2018 | 31 | 59.12 | 1.9 | 31 | 62.95 | 1.9 | 2or3or4times a week |
| Kim J.O | 2012 | 26 | 91.23 | 16.93 | 26 | 110.77 | 19.62 | 2or3or4times a week |
| Varaei S | 2020 | 32 | 4 | 1.67 | 32 | 6.22 | 1.36 | 2or3or4times a week |
| Hassanzadeh M | 2018 | 35 | 3.64 | 0.79 | 35 | 6.21 | 1.29 | once or twice a day |
| Genç F | 2020 | 30 | 3.77 | 0.68 | 29 | 4.66 | 1.33 | once or twice a day |
| Shirzadegan R. | 2020 | 40 | 24.4 | 15.75 | 40 | 71.14 | 35.25 | once or twice a day |
| Jessie H | 2019 | 21 | 0.9 | 0.76 | 20 | 1.79 | 1.63 | once or twice a day |
| [Hur M.-H.](mailto:wowmhhur@nate.com) | 2019 | 31 | 5.52 | 1.36 | 31 | 7.1 | 1.64 | once or twice a day |
| Abdollahi F. | 2020 | 30 | 63 | 3.93 | 30 | 56.26 | 4.28 | once or twice a day |
| Kyoko A | 2018 | 115 | 8.7 | 6.6 | 114 | 8.6 | 7.5 | under several hours |
| Vaziri F | 2017 | 29 | 21.68 | 14.3 | 27 | 38.22 | 15.14 | under several hours |

| **Data table 5 Subgroup analysis of treatment duration** | | | | | | | | |
| --- | --- | --- | --- | --- | --- | --- | --- | --- |
| author | year | Tsample | Tmean | Tsd | Csample | Cmean | Csd | duration |
| Demirba B.C | 2014 | 54 | 4.55 | 0.81 | 54 | 5.66 | 0.77 | ≥6w |
| Gok Metin Z | 2016 | 17 | 2.94 | 1.13 | 17 | 4.41 | 1.79 | ≥6w |
| Xu | 2020 | 49 | 39.85 | 2.64 | 49 | 33.84 | 2.17 | ≥6w |
| Kim J.O | 2012 | 26 | 91.23 | 16.93 | 26 | 110.77 | 19.62 | ≥6w |
| Varaei S | 2020 | 32 | 4 | 1.67 | 32 | 6.22 | 1.36 | ≥6w |
| Karadag E | 2019 | 30 | 35.23 | 5.21 | 30 | 38.46 | 9.12 | ≥4w |
| Sharare A | 2019 | 30 | 30.27 | 13.88 | 30 | 34.7 | 15.09 | ≥4w |
| Bagheri-Nesami M | 2016 | 29 | 42.61 | 3.45 | 30 | 41.7 | 3.39 | ≥4w |
| Hassanzadeh M | 2018 | 35 | 3.64 | 0.79 | 35 | 6.21 | 1.29 | ≥4w |
| Genç F | 2020 | 30 | 3.77 | 0.68 | 29 | 4.66 | 1.33 | ≥4w |
| Mohammadpourhodki R | 2021 | 35 | 35.4 | 15.7 | 35 | 45.1 | 14.1 | ≥4w |
| Fariba Kabiri | 2018 | 31 | 59.12 | 1.9 | 31 | 62.95 | 1.9 | ≥4w |
| Jessie H | 2019 | 21 | 0.9 | 0.76 | 20 | 1.79 | 1.63 | 2-4w |
| [Hur M.-H.](mailto:wowmhhur@nate.com) | 2019 | 31 | 5.52 | 1.36 | 31 | 7.1 | 1.64 | 2-4w |
| Shirzadegan R. | 2020 | 40 | 24.4 | 15.75 | 40 | 71.14 | 35.25 | ≤1w |
| Abdollahi F. | 2020 | 30 | 63 | 3.93 | 30 | 56.26 | 4.28 | ≤1w |
| Kyoko A | 2018 | 115 | 8.7 | 6.6 | 114 | 8.6 | 7.5 | ≤1d |
| Vaziri F | 2017 | 29 | 21.68 | 14.3 | 27 | 38.22 | 15.14 | ≤1d |

| **Data table 6 Subgroup analysis of control intervention** | | | | | | | | |
| --- | --- | --- | --- | --- | --- | --- | --- | --- |
| author | year | Tsample | Tmean | Tsd | Csample | Cmean | Csd | control |
| Karadag E | 2019 | 30 | 35.23 | 5.21 | 30 | 38.46 | 9.12 | no application |
| Bagheri-Nesami M | 2016 | 29 | 42.61 | 3.45 | 30 | 41.7 | 3.39 | no application |
| Kawabata N | 2020 | 27 | 19.6 | 19.7 | 30 | 31.8 | 26.6 | no application |
| Hassanzadeh M | 2018 | 35 | 3.64 | 0.79 | 35 | 6.21 | 1.29 | no application |
| Genç F | 2020 | 30 | 3.77 | 0.68 | 29 | 4.66 | 1.33 | no application |
| Demirba B.C | 2014 | 54 | 4.55 | 0.81 | 54 | 5.66 | 0.77 | no application |
| Gok Metin Z | 2016 | 17 | 2.94 | 1.13 | 17 | 4.41 | 1.79 | no application |
| Fariba Kabiri | 2018 | 31 | 59.12 | 1.9 | 31 | 62.95 | 1.9 | no application |
| Kyoko A | 2018 | 115 | 8.7 | 6.6 | 114 | 8.6 | 7.5 | no application |
| [Hur M.-H.](mailto:wowmhhur@nate.com) | 2019 | 31 | 5.52 | 1.36 | 31 | 7.1 | 1.64 | no application |
| Xu | 2020 | 49 | 39.85 | 2.64 | 49 | 33.84 | 2.17 | no application |
| Kim J.O | 2012 | 26 | 91.23 | 16.93 | 26 | 110.77 | 19.62 | no application |
| Varaei S | 2020 | 32 | 4 | 1.67 | 32 | 6.22 | 1.36 | no application |
| Abdollahi F. | 2020 | 30 | 63 | 3.93 | 30 | 56.26 | 4.28 | no application |
| Sharare A | 2019 | 30 | 30.27 | 13.88 | 30 | 34.7 | 15.09 | placebo |
| Shirzadegan R. | 2020 | 40 | 24.4 | 15.75 | 40 | 71.14 | 35.25 | placebo |
| Jessie H | 2019 | 21 | 0.9 | 0.76 | 20 | 1.79 | 1.63 | placebo |
| Vaziri F | 2017 | 29 | 21.68 | 14.3 | 27 | 38.22 | 15.14 | placebo |
| Mohammadpourhodki R | 2021 | 35 | 35.4 | 15.7 | 35 | 45.1 | 14.1 | massage |

| **Data table 7 Subgroup analysis of outcomes measurement** | | | | | | | | |
| --- | --- | --- | --- | --- | --- | --- | --- | --- |
| author | year | Tsample | Tmean | Tsd | Csample | Cmean | Csd | measurement |
| Karadag E | 2019 | 30 | 35.23 | 5.21 | 30 | 38.46 | 9.12 | FSS |
| Sharare A | 2019 | 30 | 30.27 | 13.88 | 30 | 34.7 | 15.09 | FSS |
| Bagheri-Nesami M | 2016 | 29 | 42.61 | 3.45 | 30 | 41.7 | 3.39 | FSS |
| Genç F | 2020 | 30 | 3.77 | 0.68 | 29 | 4.66 | 1.33 | FSS |
| Demirba B.C | 2014 | 54 | 4.55 | 0.81 | 54 | 5.66 | 0.77 | FSS |
| Gok Metin Z | 2016 | 17 | 2.94 | 1.13 | 17 | 4.41 | 1.79 | FSS |
| Mohammadpourhodki R | 2021 | 35 | 35.4 | 15.7 | 35 | 45.1 | 14.1 | FSS |
| Shirzadegan R. | 2020 | 40 | 24.4 | 15.75 | 40 | 71.14 | 35.25 | MFI |
| Fariba Kabiri | 2018 | 31 | 59.12 | 1.9 | 31 | 62.95 | 1.9 | MFI |
| Jessie H | 2019 | 21 | 0.9 | 0.76 | 20 | 1.79 | 1.63 | MFI |
| Kawabata N | 2020 | 27 | 19.6 | 19.7 | 30 | 31.8 | 26.6 | BFI |
| Hassanzadeh M | 2018 | 35 | 3.64 | 0.79 | 35 | 6.21 | 1.29 | BFI |
| Vaziri F | 2017 | 29 | 21.68 | 14.3 | 27 | 38.22 | 15.14 | VAS |
| Abdollahi F. | 2020 | 30 | 63 | 3.93 | 30 | 56.26 | 4.28 | VAS |
| Varaei S | 2020 | 32 | 4 | 1.67 | 32 | 6.22 | 1.36 | RFS |
| [Hur M.-H.](mailto:wowmhhur@nate.com) | 2019 | 31 | 5.52 | 1.36 | 31 | 7.1 | 1.64 | NRS |
| Xu | 2020 | 49 | 39.85 | 2.64 | 49 | 33.84 | 2.17 | FSAS |
| Kim J.O | 2012 | 26 | 91.23 | 16.93 | 26 | 110.77 | 19.62 | FAI |
| Kyoko A | 2018 | 115 | 8.7 | 6.6 | 114 | 8.6 | 7.5 | Checklist for Assessment of Worker’s Accumulated Fatigue |

| **Data table 8 Subgroup analysis of type of population** | | | | | | | | |
| --- | --- | --- | --- | --- | --- | --- | --- | --- |
| author | year | Tsample | Tmean | Tsd | Csample | Cmean | Csd | type of population |
| Karadag E | 2019 | 30 | 35.23 | 5.21 | 30 | 38.46 | 9.12 | hemodialysis treatment |
| Sharare A | 2019 | 30 | 30.27 | 13.88 | 30 | 34.7 | 15.09 | hemodialysis treatment |
| Bagheri-Nesami M | 2016 | 29 | 42.61 | 3.45 | 30 | 41.7 | 3.39 | hemodialysis treatment |
| Hassanzadeh M | 2018 | 35 | 3.64 | 0.79 | 35 | 6.21 | 1.29 | hemodialysis treatment |
| Varaei S | 2020 | 32 | 4 | 1.67 | 32 | 6.22 | 1.36 | hemodialysis treatment |
| Mohammadpourhodki R | 2021 | 35 | 35.4 | 15.7 | 35 | 45.1 | 14.1 | hemodialysis treatment |
| Gok Metin Z | 2016 | 17 | 2.94 | 1.13 | 17 | 4.41 | 1.79 | Arthritis |
| Fariba Kabiri | 2018 | 31 | 59.12 | 1.9 | 31 | 62.95 | 1.9 | Arthritis |
| Kawabata N | 2020 | 27 | 19.6 | 19.7 | 30 | 31.8 | 26.6 | cancer |
| Xu | 2020 | 49 | 39.85 | 2.64 | 49 | 33.84 | 2.17 | cancer |
| [Hur M.-H.](mailto:wowmhhur@nate.com) | 2019 | 31 | 5.52 | 1.36 | 31 | 7.1 | 1.64 | blood glucose abnormality |
| Abdollahi F. | 2020 | 30 | 63 | 3.93 | 30 | 56.26 | 4.28 | blood glucose abnormality |
| Kyoko A | 2018 | 115 | 8.7 | 6.6 | 114 | 8.6 | 7.5 | women after dilivery |
| Vaziri F | 2017 | 29 | 21.68 | 14.3 | 27 | 38.22 | 15.14 | women after dilivery |
| Demirba B.C | 2014 | 54 | 4.55 | 0.81 | 54 | 5.66 | 0.77 | female with fibromyalgia |
| Shirzadegan R. | 2020 | 40 | 24.4 | 15.75 | 40 | 71.14 | 35.25 | acute myocardial infarction |
| Jessie H | 2019 | 21 | 0.9 | 0.76 | 20 | 1.79 | 1.63 | women with hypothyroidism |
| Genç F | 2020 | 30 | 3.77 | 0.68 | 29 | 4.66 | 1.33 | institutionalized elderly |
| Kim J.O | 2012 | 26 | 91.23 | 16.93 | 26 | 110.77 | 19.62 | women in rural areas |

| **Data table 9** Sensitivity analysis | | | | | | | |
| --- | --- | --- | --- | --- | --- | --- | --- |
| author | year | Tsample | Tmean | Tsd | Csample | Cmean | Csd |
| Hassanzadeh M | 2018 | 35 | 3.64 | 0.79 | 35 | 6.21 | 1.29 |
| Genç F | 2020 | 30 | 3.77 | 0.68 | 29 | 4.66 | 1.33 |
| Demirba B.C | 2014 | 54 | 4.55 | 0.81 | 54 | 5.66 | 0.77 |
| Gok Metin Z | 2016 | 17 | 2.94 | 1.13 | 17 | 4.41 | 1.79 |
| Mohammadpourhodki R 1 | 2021 | 35 | 35.4 | 15.7 | 35 | 45.1 | 14.1 |
| Shirzadegan R. | 2020 | 40 | 24.4 | 15.75 | 40 | 71.14 | 35.25 |
| Fariba Kabiri | 2018 | 31 | 59.12 | 1.9 | 31 | 62.95 | 1.9 |
| Jessie H | 2019 | 21 | 0.9 | 0.76 | 20 | 1.79 | 1.63 |
| [Hur M.-H.](mailto:wowmhhur@nate.com) | 2019 | 31 | 5.52 | 1.36 | 31 | 7.1 | 1.64 |
| Vaziri F | 2017 | 29 | 21.68 | 14.3 | 27 | 38.22 | 15.14 |
| Kim J.O | 2012 | 26 | 91.23 | 16.93 | 26 | 110.77 | 19.62 |
| Varaei S | 2020 | 32 | 4 | 1.67 | 32 | 6.22 | 1.36 |
